# Exposure, perceived risk, and psychological distress among general population during the COVID-19 lockdown in Wuhan, China

**DOI:** 10.3389/fpsyt.2023.1086155

**Published:** 2023-04-14

**Authors:** Yujun Liu, Linping Liu, Zhilei Shi

**Affiliations:** ^1^Department of Social Work and Social Policy, School of Social and Behavioral Sciences, Nanjing University, Nanjing, Jiangsu Province, China; ^2^Department of Sociology, School of Social and Behavioral Sciences, Nanjing University, Nanjing, Jiangsu Province, China; ^3^Center for Population and Health Research, Faculty of Public Administration, Zhongnan University of Economics and Law, Wuhan, Hubei Province, China

**Keywords:** COVID-19, psychological distress, exposure, perceived risk, general population, Wuhan lockdown

## Abstract

**Introduction:**

The COVID-19 pandemic that has been going on since the end of 2019 impacts people on both the physical and psychological levels. However, the psychological status, especially its underlying psychosocial mechanisms among the general population in Wuhan, the earliest epicenter and hardest-hit city in China during the pandemic, has not been well investigated. This study aimed to examine the relationships between exposures, perceived risk, and psychological distress among the general population in Wuhan during the COVID-19 lockdown.

**Methods:**

Data were from a cross-sectional online survey conducted from 20 February to 4 March 2020. Final analyses included 4,234 Wuhan respondents. A 5-item Hopkins Symptom Checklist was adopted to assess respondents’ psychological distress.

**Results:**

It was found that nervousness, fear, and worry were the most common symptoms among Wuhan residents during the lockdown. Exposure within a close physical distance, exposure within the social network, and perceived risk are significantly positively related to respondents’ psychological distress. Moreover, perceived risk mediated the effects of exposures on respondents’ psychological condition.

**Discussion:**

These findings conduce to identify the populations at higher risk of suffering psychological disturbance during the pandemic and are expected to inform the policymakers and mental health professionals to monitor and improve the perception of risk among the target population by appropriate interventions.

## Introduction

Since the first batch of cases was confirmed in late December 2019 in Wuhan, the provincial capital of Hubei Province in China, the impact of Coronavirus Disease 2019 (COVID-19) has been ongoing for more than 3 years. Currently, there have been more than 750 million confirmed cases and over 6.8 million deaths reported to WHO (1).

From the outbreak in Wuhan, the Chinese government undertook unprecedented measures, including travel restrictions and quarantines to reduce and prevent transmission (2). Wuhan, a city with more than 13 million regular residents, implemented a 76-day lockdown from 23 January to 8 April 2020. Such strict control measures were certified to efficiently contain the virus (3, 4). To date, there is considerable research into the psychological consequences caused by this precipitate pandemic and relevant responses, among which the mental health of frontline healthcare workers and vulnerable groups (like college students) received sufficient attention (5, 6). However, very few studies have focused on the mental wellbeing of the general population in Wuhan during the lockdown (7). Under this circumstance, the present study attempted to (1) describe the psychological distress experienced by the general population in Wuhan; and (2) explore the potential psychosocial mechanism leading to distress. Findings are expected to facilitate the identification of high-risk groups and the provision of effective mental health services in cities with large populations like Wuhan when confronting a similar emergency pandemic.

### COVID-19 pandemic and its psychological impacts

Instant and long-term mental health concerns are common responses to the COVID-19 pandemic (8, 9). Two meta-analyses revealed that the pandemic increases the prevalence of stress, depression, anxiety, and insomnia (10, 11). COVID-19 patients, patients with preexisting psychiatric symptoms, and healthcare workers were found to suffer from poorer mental health conditions (12). In general population, female gender, younger age, chronic illnesses, unemployment, student status, and frequent exposure to social media/news concerning COVID-19 were significant correlates linked to mental distress (13). Psychological resources, such as personalized psychological flexibility, resilience, and extraversion were found to act as mediators when explaining the psychological distress among the general population (14, 15). In summary, descriptive epidemiological studies on this topic are predominant, while research on specific psychosocial mechanisms behind mental health problems, especially the interaction between objective and subjective factors among the general population during this pandemic is in its infancy (12, 16).

In China, numerous studies are carried out since the Wuhan outbreak to facilitate mental health treatment and care during the pandemic (17, 18). Despite the various sample size and measuring tools, these studies reached a consensus that mental health symptoms were common among the general population in China during the COVID-19 outbreak. Individuals’ characteristics, such as gender, age, education, occupation, and geographical location were found to be significantly associated with mental wellbeing (19–22). In particular, residents in Hubei Province had increased odds of mental health symptoms such as depression, anxiety, and posttraumatic stress symptoms (23–25). Nonetheless, there are hardly any studies focused on the psychological status of the general population living in Wuhan, who were exposed to a huge risk of infection in the earliest epicenter of the COVID-19 outbreak in China.

### Exposure to COVID-19 and psychological distress

COVID-19 is an infectious disease that is easily transmitted and rapidly spreading. That means, exposure to COVID-19, no matter by contacting with an infected object or environment, or by human-to-human approach in a family or community, has a degree of risk of getting infected (26). Existing evidence suggested that exposure to infectious diseases of the respiratory system is closely associated with individuals’ mental health outcomes, among which the frontline healthcare practitioners obtained more academic attention (27). For instance, Wu et al. (28) revealed that hospital employees who worked in high-risk locations or had friends or close relatives who were contracted, were more likely to exhibit posttraumatic stress symptoms during the SARS epidemic in Beijing, China. In a Korean study, medical staff who were engaged in MERS-related tasks had highest risk to exhibit post-traumatic stress disorder symptoms (29).

Jiang et al. (30) conducted a similar study among the general population in China. They cited an official guiding principle of emergent psychological crisis intervention and divided the participants recruited by social media into four groups. The participants ranged from level 1 (patients with severe symptoms of COVID-19, frontline medical workers, CDC researchers, or administrative staff) to level 4 (people in affected areas, susceptible groups, or the general public). They found that exposure level was significantly linked to participants’ post-traumatic stress symptoms (PTS) only in the mildly PTS symptom subgroup, rather than the moderate and high PTS symptoms subgroups.

As suggested in prior studies, exposure to infectious diseases such as COVID-19 is uneven across different populations. Social and environmental factors contribute to shaping people’s exposure risk (31). In addition to the occupational factor mentioned earlier, geographical location is also closely related to the individual’s exposure. In China, Wuhan is the first city that reported confirmed cases of COVID-19 at the end of 2019, most of which had exposure history of Huanan Seafood Wholesale Market. On 20 January, the Wuhan government declared that a headquarters of epidemic prevention and control was set up. On 23 January, the headquarters announced that channels to leave Wuhan by airport or railway stations would be temporarily closed, and the residents should not leave Wuhan if not necessary (32). As of 24:00 on 9 February, there were a total of 35,982 confirmed cases in China, of which 16,902 cases were reported in Wuhan (33). On 10 February, the headquarters proclaimed that closed-off management would be carried out among all of the housing estates in Wuhan to reduce the contact of people and interrupt the transmission of COVID-19 (34). In the meantime, residents were required to stay at home and avoid visiting public places. Residential buildings which had confirmed or suspected cases of COVID-19 were strictly monitored and managed.

Although these strict measures were implemented in succession and the occupational exposure among the general population was far less than that among healthcare workers, residents inevitably experienced other forms of exposure to COVID-19 outbreak events, such as having a family member or friend who was infected. In a study based on the nationwide sample, 1.1% of the respondents had at least one family member or friend who was infected with COVID-19 (25). In an online study conducted among the general population in Hubei Province (Wuhan residents accounted for nearly half of the sample), there were 17.9% of the respondents who were familiar with someone who had COVID-19, of which three quarters revealed that a familiar relationship was friendship (23). Accordingly, we believed that this situation was not uncommon among Wuhan residents, since there were approximately half of the confirmed cases across the country reported in Wuhan at that time, suggesting a necessity to examine its psychological impact.

Thus far, little is known about the mechanism between exposure and psychological status among the general population in Wuhan, the hardest-hit city in China. In addition, exposure to COVID-19 has different levels, which are not fully addressed in the most existing literature. Given that the general population were required to stay at home and strictly keep social distancing during the Wuhan lockdown, exposure could be divided into two categories: exposure within a certain physical distance and exposure within the social network. The former refers to having a family member living together, a neighbor living in the same building, or a resident living in the same housing estate, who contracted COVID-19. The latter includes having family members not living together or people in the social network (such as friends, colleagues, and acquaintances) who contracted COVID-19. In other words, exposure at a certain physical distance means a degree of direct infection risk, while exposure in the social network indicates the possible prevalence of the disease. Because COVID-19 is highly infectious, exposure in or out of a certain physical distance might have different impacts on individuals’ psychological status at the beginning of the outbreak, when the knowledge of the virus and medical materials were extremely limited. To distinguish different levels of exposure among the general population in Wuhan and evaluate their impacts, we propose the first set of hypotheses as follows:

*Hypothesis 1a*: Exposure to COVID-19 within a close physical distance is positively associated with psychological distress among the general population in Wuhan.

*Hypothesis 1b*: Exposure to COVID-19 within the social network is positively associated with psychological distress among the general population in Wuhan.

### Perceived risk of COVID-19 and psychological distress

In addition to the objective exposure experiences, people’s attitudes, feelings, and perceptions are also closely related to their psychological outcomes when confronting different kinds of crisis events (35–37). Risk perception refers to people’s subjective judgments about the likelihood of negative occurrences, including injury, illness, disease, and death (38). There is a body of work suggesting that perceived risk is a salient factor linked to mental health outcomes among healthcare workers or the general population during some infectious disease outbreaks. A systematic and thematic review demonstrated that perceived risk was significantly linked to the psychological wellbeing among healthcare employees during the SARS crisis (39). Jalloh et al. (40) revealed that risk perception was independently associated with anxiety-depression and PTSD symptoms in the general population in an African country affected by the Ebola pandemic.

A handful of existing studies examined the relations between the perceived risk of COVID-19 and an individual’s mental health. Yıldırım et al. (41) found that perceived risk positively predicted depression, anxiety, and stress among 204 healthcare professionals who were actively treating patients confirmed with COVID-19 in Turkey. Kim et al. (42) indicated that a higher perceived risk of COVID-19 infection was associated with greater depressive symptoms during the first 6 weeks of quarantine in 221 adults in urban South Africa. Dratva et al. (43) reported that the perceived risk of COVID-19 was significantly associated with general anxiety among Swiss university students.

Similar studies were conducted among the Chinese population, all of which utilized the online questionnaire survey with a sample size ranging from 693 to 2,993 (44–46). The perceived risk of COVID-19 measured by one or more proxies, such as perceived severity, perceived controllability, perceived risk of infection, affective risk perception, or cognitive risk perception, was revealed to significantly relate to individuals’ mental health in existing studies. However, scant attention had been paid to Wuhan residents, who lived in the epicenter of China at the beginning of the pandemic. At that time, the origin of the virus, patterns of spread, therapeutic strategy, and the prognosis of the disease remained unclear. In addition, unprecedented strict measures such as lockdown and closed-off management of intra-city housing estates were implemented consecutively, and more than 30,000 medical practitioners from all over the country were sent to assist the medical system in Wuhan. How Wuhan residents perceive this pandemic in such an uncertain environment and how their perceived risk relates to psychological status have not been fully addressed in previous studies. Therefore, we develop the second hypothesis as follows:

*Hypothesis 2*: Perceived risk is positively related to psychological distress among the general population in Wuhan.

Furthermore, although people confront the same situation during a crisis like the COVID-19 pandemic, they perceive it in different ways due to knowledge, certainty about the risk, or even individuals’ characteristics. Paek and Hove (38) gave an example in the health context, indicating that people are more likely to perceive colon cancer as a highly fatal disease if they have friends or family members who died of it. This point is echoed by a study on the 2003 SARS outbreak in Beijing, indicating that hospital employees who experienced work exposure or any quarantining perceived a greater level of risk, and the perceived risk was significantly associated with PTS symptom level (28). Such mediating effects of individuals’ subjective perception between the objective exposure experience and mental health outcome, demonstrated in the aforementioned study, were rarely examined among the existing studies on COVID-19. Having a family member, relative, friend, or neighbor who was infected with COVID-19 was not infrequent among the general population in Wuhan. Whether such exposure experience shapes residents’ perceived risk and thereby indirectly affects their mental health status needs to be further elucidated. Furthermore, people may have different perceptions of risk when they encounter different levels of exposure. Therefore, drawing lessons from the previous empirical evidence, we propose the third set of hypotheses:

*Hypothesis 3a*: Perceived risk mediates the relationship between exposure to COVID-19 at a certain physical distance and psychological distress among the general population in Wuhan.

*Hypothesis 3b*: Perceived risk mediates the relationship between exposure to COVID-19 in the social network and psychological distress among the general population in Wuhan.

To sum up, this study had three goals: to evaluate the psychological distress among the general population during the Wuhan lockdown; to explore the relations between two levels of exposure to the COVID-19 outbreak and psychological distress; and to examine the mediating effect of perceived risk between exposure and psychological distress. To the best of our knowledge, this study was one of the first attempts to investigate the psychological impacts of COVID-19 based on a relatively large Wuhan sample.

## Methods

### Research design and sample

Data were retrieved from the Community Life Survey among General Population in Wuhan during the COVID-19 Outbreak, which was jointly carried out by researchers at the Zhongnan University of Economics and Law, the Hong Kong University of Science and Technology, and the Huazhong University of Science and Technology. The formal survey was conducted from 20 February to 4 March 2020, around a month after Wuhan implementing lockdown. The survey was implemented in three steps. First, we issued online recruitment notices in colleges and universities in Wuhan, setting criteria for investigators: adult and living in Wuhan at that time. More than 300 students submitted the entry form. We interviewed them and finally selected 149 investigators covering all 13 administrative districts in Wuhan, during which we considered the population size of each district. Second, a pilot study was carried out from 10 to 19 February, according to which the questionnaire was refined and finalized. Then, the investigators were strictly trained in the online meeting to ensure that they could offer the necessary assistance to their respondents.

The sampling approach of this online survey differed from most similar studies conducted simultaneously in China, that is, eligible respondents were accessed by investigators proactively. Several inclusion criteria about the respondents were set in advance: lived in Wuhan during the COVID-19 outbreak; one respondent in one household (household was defined by the independent right of property); and gender balance as much as possible. We required investigators to note the age structure of potential respondents. For instance, respondents should cover youth, middle-aged people, and old people. We did not prescribe any limits to the personal relationship between the investigator and his/her interviewees.

An online questionnaire was adopted in this survey. Each investigator was assigned a personal account so that the questionnaire that responded under this account could be easily tracked. To ensure the authenticity of the survey and the data quality, we forbade the investigators to share the questionnaire link on social network platforms (such as WeChat group and WeChat moment which were widely used in China) and required them to communicate with each potential respondent by communication tools (such as telephone and WeChat) before sending the questionnaire. Respondents were informed of the objective and the content of this survey. The website link and password of the questionnaire were sent to the respondents after obtaining their consent. Respondents were asked to directly submit the questionnaire online once completed. Every investigator was required to send out at least 15 questionnaires, but we did not propose an upper limit on the number of questionnaires for every investigator. We terminated the survey when the sample covered all districts in Wuhan, with 4,267 completed questionnaires. To examine the quality of the survey, we randomly selected some respondents and paid a return visit to them by telephone. In the final analyses, a total of 4,234 respondents were included. This study was approved by the Ethics Committee of Zhongnan University of Economics and Law.

### Measures

#### Psychological distress

We assessed the respondents’ psychological distress using the 5-item Hopkins Symptom Checklist (HSCL-5), which was a screening instrument to measure the symptoms of anxiety and depression (47, 48). Respondents were required to describe how often they have felt nervous/fearful/blue/worried too much/hopeless about the future during the past week. Five response options, including rarely or none of the time (less than 1 day), some or a little of the time (1–2 days), occasionally or a moderate amount of time (3–4 days), frequently or most of the time (5–6 days), and all of the time (7 days), were scored 1 to 5 successively. We totaled up the score of each item and the higher total score indicated a higher level of psychological distress. HSCL-5 in this study had good reliability and the Cronbach’s alpha was 0.9139.

#### Exposure to the COVID-19 outbreak

Respondents were asked if there was anyone around them infected with COVID-19 by five questions. We established two proxies to indicate two types of COVID-19 outbreak event exposures. One was the exposure within a close physical distance if respondents’ kinsfolk who was living together, resident who lived in the same building, or resident who lived in the same housing estate were infected (no = 0, yes = 1); another was the exposure within the social network, indicating respondents’ kinsfolk who was not living together or friend/schoolmate/colleague were infected (no = 0, yes = 1).

#### Perceived risk

Given that COVID-19 is highly contagious, we adopted a single question to assess respondents’ perception of pandemic-related risk: “Are you worried about yourself getting infected with COVID-19″, on a 5-point Likert scale, ranging from 1 (not at all worried) to 5 (extremely worried). Perceived risk was considered a continuous variable in subsequent analysis. We utilized mean value imputation to deal with its slight missing data (*N* = 4,175).

#### Socio-demographic characteristics

Respondents’ socio-demographic information, including gender, age, education level, marriage, employment status, and self-report family social economic status (SES), was obtained in the survey.

### Statistical analyses

Random iterative method (RIM) weighting was utilized to deal with the potential sampling bias. RIM weighting allows researchers to weigh each variable as an individual entity to ensure that each data point is accurately represented while keeping the characteristics proportionate as a whole (49). Given that the investigators in our online survey were students from colleges and universities, the unweighted sample was relatively young and well-educated. Weight factors were generated and applied to the sample such that the weighted sample matched the two independent distributions, that is, age and education status (50). Subsequent analyses were conducted in weighted data.

We used descriptive analyses to summarize the characteristics of the sample. T-tests and ANOVA were adopted to examine whether psychological distress was different across disparate groups in one category. Pairwise comparisons of HSCL-5 mean score between any two groups in one category were conducted using the Scheffe approach (51). Then, we performed the multivariate linear regressions to explore the relations among exposures, perceived risk, and psychological distress. Models 1 and 2 examined the effects of exposure within a close physical distance and within the social network on psychological distress, respectively, and Model 3 simultaneously adopted two kinds of exposures. Finally, we employed the mediating effect analyses to investigate the mediating effect of perceived risk between exposures and psychological distress. Sobel–Goodman tests were utilized to examine whether the mediating effects were significant (52, 53).

## Results

The mean total score of HSCL-5 was 11.95 (SD = 5.51). Nervousness, fear, and excessive worry were found to be the most common symptoms among Wuhan residents. There were 11.81% and 10.42% of the respondents who felt nervous and fearful all of the time. Although confronting the unprecedentedly tough situation, more than half of the respondents (54.25%) did not feel hopeless about the future.

Table 1 shows the respondents’ socio-demographic characteristics. Of the sample, there were more female than male respondents. The mean age was 37.17 (SD = 14.66). More than half of the respondents held a bachelor’s or above degree. Nearly 60% of the respondents were married; 51.61% of the respondents were at work and 11.93% of the respondents were out of work. Half of the respondents regarded that their family SES was on the middle level. With respect to the explanatory variables, almost 70% of the respondents confirmed that they were confronting the exposure within a close physical distance, and nearly 40% of the respondents reported that they were encountering the exposure within the social network. The overwhelming majority of the respondents were worried about themselves getting infected with COVID-19.

**Table 1:** Descriptive statistics of individual characteristics and psychological distress in different categories among Wuhan residents (*N* = 4,234).

	N (%)	*p* value	Mean score of HSCL-5
*Gender*			
Male	1821 (43.01)	0.000	11.34
Female	2,413 (56.99)		12.41
*Age*			
< 30	1,570 (37.08)	0.006	11.27_a_
30–60	2077 (49.06)		12.39_b_
>60	587 (13.86)		12.21_b_
*Education*			
Primary school or below	55 (1.30)	0.151	12.56
Junior secondary school	377 (8.90)		12.11
Senior secondary school	750 (17.71)		12.21
Junior college	884 (20.88)		12.07
Bachelor degree or above	2,168 (51.20)		11.77
*Marriage*			
Unmarried	1,580 (37.32)	0.001	11.30_a_
Married	2,438 (57.58)		12.33_b_
Others	216 (5.10)		12.43_b_
*Employment status*			
In work	2,185 (51.61)	0.002	12.17_a_
Out of work	505 (11.93)		13.15_b_
Student	1,126 (26.59)		11.03_c_
Retirement	418 (9.87)		11.85_a_
*Self-report family SES*			
Below the middle level	1806 (42.65)	0.004	12.54_a_
Middle level	2,117 (50.00)		11.53_b_
Above the middle level	311 (7.35)		11.41_b_
*Exposure within close physical distance*			
Yes	2,943 (69.51)	0.000	12.31
No	1,291 (30.49)		11.13
*Exposure within social network*			
Yes	1,618 (38.21)	0.000	12.64
No	2,616 (61.79)		11.53
*Perceived risk*			
Not at all worried	589 (14.11)	0.000	9.29_a_
Slightly worried	767 (18.37)		10.93_b_
Moderately worried	1,430 (34.25)		11.74_c_
Very worried	614 (14.71)		12.61_d_
Extremely worried	775 (18.56)		14.66_e_

*p*-values in the third column were obtained by *T*-test (for binary variables: gender and two exposure factors) and ANOVA (for multinomial variables). Subscripted lowercase letters (a, b, c, d, e) in the fourth column represented the results of pairwise comparisons of the mean total score of HSCL-5. The same letters meant that the difference between any two disparate groups in one category was not statistically significant, while the different letters meant significant differences at 0.001, 0.01, or 0.05 levels.

Table 1 also presents the mean score of HSCL-5 in different categories among respondents. *p*-values showed that except for the education status, the remaining five socio-demographic factors and three explanatory factors were significantly associated with a mean score of HSCL-5. Results of pairwise comparisons demonstrated the significant differences between any two groups in one category. For instance, the HSCL-5 mean scores of respondents who were in work were significantly lower than respondents who were out of work, while the mean scores of respondents who were in work did not significantly differ from respondents who were retired. The mean score of respondents whose family SES was middle level was not significantly different from that of respondents whose SES was above the middle level. Education was not included in subsequent regression analyses, because its correlation with respondents’ psychological distress was not significant, it was not included in subsequent regression analyses. Two exposure-related variables and perceived risk were significantly linked to respondents’ psychological distress, indicating the necessity to conduct further regression analysis.

Table 2 shows the results of three regression models, controlling for gender, age, marriage, employment status, and self-report family SES. Model 1 was the psychological distress regressed on exposure within a close physical distance and perceived risk, Model 2 was the psychological distress regressed on exposure within the social network and perceived risk, and Model 3 included two types of exposures and perceived risk. Regression results showed that female respondents were more likely to suffer psychological distress than male respondents. Compared to respondents in work, respondents who were out of work in our sample were more likely to experience psychological distress. Students had better mental health status, which was significant on the 0.1 level. Retirees also had a lower risk of suffering psychological distress compared to respondents in work, although it was not statistically significant. Compared to the respondents who regarded their families as below the middle level in society, the mental health condition of respondents who regarded their families as the middle level or above was significantly better. Age group and marriage were not significantly linked to respondents’ psychological distress.

**Table 2:** Results of three multivariate linear regressions (*N* = 4,234).

	Model 1	Model 2	Model 3	Model 3
	Coefficient	Coefficient	Coefficient	Standardized coefficient
*Gender (Male = 0)*				
Female	0.9517***	0.9622***	0.9387***	0.0845***
*Age (<30 = 0)*				
30–60	0.4179	0.4024	0.3631	0.0330
>60	0.4541	0.4576	0.4342	0.0272
*Marriage (Unmarried = 0)*				
Married	−0.0508	−0.0276	−0.0374	−0.0034
Others	0.0580	0.0072	0.0403	0.0016
*Employment (In work = 0)*				
Out of work	0.8110**	0.7970**	0.8771**	0.0516**
Student	−0.5473^#^	−0.5065^#^	−0.5017^#^	−0.0404^#^
Retirement	−0.4199	−0.4237	−0.4295	−0.0232
*Self-report family SES (Below the middle level = 0)*				
Middle level	−0.7610***	−0.7690***	−0.7987***	−0.0727***
Above the middle level	−0.6901*	−0.6509*	−0.7247*	−0.0345*
*Exposure within close physical distance (No = 0)*				
Yes	0.8712***		0.7210***	0.0605***
*Exposure within social network (No = 0)*				
Yes		0.9033***	0.7782***	0.0687***
*Perceived risk*	1.1530***	1.1596***	1.1390***	0.2651***
Constant	7.5309***	7.7544***	7.4152***	
Adjusted *R*^2^	0.1087	0.1098	0.1130	0.1130
Variance inflation factor	1.93	1.93	1.87	1.87

*** *p* < 0.001;

** *p* < 0.01;

* *p* < 0.05;

# *p* < 0.1.

As expected, Hypotheses 1a, 1b, and 2 were supported by empirical data. To be specific, exposures, no matter whether within a close physical distance or within the social network, were saliently linked to respondents’ psychological distress. Perceived risk was significantly positively associated with psychological distress, that is, the more worried about getting infected with COVID-19, the worse the mental health status. In Model 3, the standard coefficient of exposure within the social network was slightly larger, indicating that its effect was slightly stronger than the exposure within a close physical distance on psychological distress. Moreover, the variance inflation factor (<10) displayed in the last column of Table 2 indicated that there were no obvious collinearity problems in these regression models.

**Figure 1:**
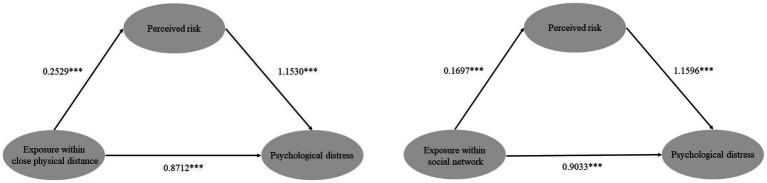
The mediating effect of perceived risk between exposure within a close physical distance/exposure within the social network and respondents’ psychological distress (***p < 0.001).

Then, we examined the mediating effects of perceived risk between exposures and respondents’ psychological distress, controlling for gender, age, marriage, employment, and self-report family SES. The main findings are displayed in Figure 1. As for the relationship between exposure within a close physical distance and psychological distress, the direct effect was 0.8712 and the indirect effect was 0.2916. The mediating effect of perceived risk was statistically significant with 25.08% of the total effect being mediated. With regard to the relationship between exposure within the social network and psychological distress, the direct effect was 0.9033 and the indirect effect was 0.1968. The mediating effect of perceived risk was of statistical significance, with 17.89% of the total effect being mediated. Hypotheses 3a and 3b were confirmed.

## Discussion

### Main findings and implications

The current study focuses on the psychological distress among the general population in Wuhan, who lived in the earliest epicenter of the COVID-19 outbreak and experienced the unprecedented lockdown. According to an online questionnaire survey, we collected valid data from 4,234 respondents. We found that nervousness, fear, and worry were the most common symptoms among Wuhan residents, while depressed feeling was relatively less prevalent. Although confronting an extremely tough situation during the early outbreak, most of the respondents did not feel hopeless about the future.

We also investigated the exposure to the COVID-19 outbreak among the general population in Wuhan, by asking them if there were anyone physically close to them or in their social network who had contracted the virus. In our sample, nearly 70% of the respondents reported that their families who lived together and the residents who lived in the same building or the same housing estate were infected. In addition, there were approximately 40% of the respondents reported that their families who were not living together or their friends/schoolmate/colleagues were infected. Even though the exposures in our study were self-reported, these findings contributed to the current knowledge of the prevalence of COVID-19 in Wuhan from the perspective of local residents. Moreover, although the social and economic consequences of this pandemic are far-reaching, people’s perceptions of the same situation are divergent. In our sample, less than 15% of the respondents were not worried about contracting the coronavirus at all, and nearly 20% of the respondents were extremely worried about getting infected.

We performed multivariate linear regressions to explore the correlates of psychological distress among Wuhan residents. Female respondents were more likely to experience psychological distress, which was consistent with the results of previous studies with a national sample, even though we utilized different measuring tools for mental health outcomes (21, 22). Compared with the respondents who were at work, those who were out of work were more vulnerable to poor mental health, which was in accordance with a previous study (54). Students had better mental health on the 0.1 level, which differed from the finding of Wang et al. (22). In the research of Wang et al. (22), student status was significantly associated with a greater psychological impact of the outbreak and higher levels of stress, anxiety, and depression. The possible reason is that the surveys were conducted at different time points and distinct measuring tools were adopted. We found that higher family SES was a protective factor of people’s mental health status during the pandemic. Those Wuhan residents who regarded their family SES as the underclass were at higher risk of psychological distress. These findings may assist policymakers in accurately identifying people who are more susceptible to psychological disturbance and providing essential mental health services to target populations during and after the pandemic in time.

As Hypotheses 1a and 1b suggested, exposure within a close physical distance and exposure within the social network were significantly associated with psychological distress among Wuhan residents independently. This result was partly in accordance with a national online survey study involving all 34 province-level regions in China, indicating that people who had a relative with confirmed or suspected COVID-19 were more likely to have negative mental health outcomes (25). However, this study did not distinguish the disparate types of exposure and failed to compare their effects consequently.

We included two types of exposures simultaneously in one linear regression model (Model 3), finding that the standard coefficient of exposure within the social network was slightly larger than that of exposure within a close physical distance. An Individual’s social network size is usually larger than the size of his/her kinship (55, 56). To some extent, more exposure within the social network meant that the infectivity and severity of COVID-19 were great and uncontrollable, which might cause more panic and distress among residents directly. Hypotheses 2, 3a, and 3b, focusing on the direct and mediating effects of perceived risk on Wuhan residents’ psychological distress, were all confirmed by empirical data. Respondents who were exposed to COVID-19 were more likely to be worried about being contracted, and those who were more worried exhibited more distress. What is noteworthy is that perceived risk mediated more of the total effect of exposure within a close physical distance on psychological distress than that of the exposure within the social network (25.08 vs. 17.89%) in our study. This finding was consistent with the contagious nature of COVID-19, which can rapidly transmit within the family and the community (57). Infected family members who were living together or infected residents who lived in the same building or the same housing estate enabled respondents to perceive a higher risk of getting infected, and then to express more distress. The mediating effect of the perceived risk between exposure within the social network and psychological distress was relatively weaker.

We argue that demonstrating the interaction between objective exposures to the COVID-19 outbreak and subjective perception of risk on psychological distress among the general population in Wuhan is a distinct contribution of the present study. Perceived risk is associated with a greater likelihood of engagement in preventive behaviors, such as wearing the mask, avoidance of public transportation, frequent handwashing, COVID-19 testing, and vaccination (58, 59). Understanding the complicated mechanisms between inevitable exposures, psychosocial factors, and an individual’s mental wellbeing during the pandemic is beneficial to increase preparedness for the unforeseeable future outbreak and other public health crises. However, given the negative impact of perceived risk on an individual’s psychological outcome, the governmental sector and healthcare institutions have obligation to apply the appropriate health communication strategy, medium, and tool to allay the fears and maintain the perceived risk at a moderate level. Diversity of exposure level and risk perception across different social groups should be taken into consideration when designing and delivering health education and crisis intervention program. Moreover, people’s exposure and risk perception vary across different stages of the pandemic, which should be fully considered by policymakers.

## Limitations and future directions

This study has the following limitations. First, our sample was obtained from the social networks of the investigators recruited from colleges and universities in Wuhan, which might explain that around 27% of the respondents were students, even if this number was much smaller than that in similar studies (60). Hence, we collected data using the web-based questionnaire survey, implying that only those residents who were accessed by our investigators and who were able to answer the questions subjectively and objectively (e.g., can use a computer or smartphone), were sampled in our survey. Unlike other similar studies, we did not release the questionnaire directly on the social network platform to assure the survey quality. We also used the Random Iterative Method weighting to minimize the sampling bias in analyses.

Although the nonrandom sampling prevented statistical inference from the sample to the whole population, our study provided valuable information about the real situation among Wuhan residents, who lived in the city that was most severely hit by this pandemic. The online survey was one of the most appropriate options to timely collect valid information in such an emergent period that the new infectious disease caused panic and anxiety widely. Second, although we found that exposure to the COVID-19 outbreak and perceived risk were significantly associated with Wuhan residents’ psychological distress, it should be cautious to establish a causal relationship due to the cross-sectional design. Large-scale longitudinal research based on random sampling is needed to address the long-term psychological impacts of this pandemic.

Last but not the least, even though the present study provided a psychosocial perspective to understand the psychological distress of Wuhan residents, a more comprehensive theoretical model and multidimensional measurements should be adopted in future work. For instance, exposure to the pandemic is diversiform, containing but not limited to occupational exposure, media exposure, and family or community sources of exposure. People may experience one or more exposures during the pandemic. Considering that the lockdown and closed-off management of housing estates were strictly implemented in Wuhan and the whole city nearly ceased to function when the survey was carried out, we merely evaluated two types of exposures among the general population (exposure within a certain physical distance vs. exposure within the social network), which were not underlined in previous studies. In addition, considering that the perceived risk of infection was of most concern during the pandemic, we enrolled it as the indicator of perceived risk. Future studies should seek to reveal the complicated mechanisms between different types of exposures, multifaceted risk perceptions, and individuals’ mental health outcomes, using more integrated statistical instruments.

Moreover, we utilized HSCL-5, an economical symptom assessment measure instead of the scales with dozens of items to evaluate the mental health consequence among Wuhan residents. Our concern was that the lengthy questionnaire might negatively affect the quality of answers during that rattled time. Although HSCL-5 had good internal consistency in the present study, combining the screening tool with the diagnostic tool to accurately assess the mental health outcome of people affected by the pandemic is of significance in future studies.

## Conclusion

In conclusion, based on the data collected by an online questionnaire survey, the present study described the psychological distress exhibited by the general population in Wuhan during the COVID-19 lockdown. Nervousness, fear, and worry were found to be common among Wuhan residents. More importantly, the present study was among the first to elucidate the underlying mechanism between COVID-19 exposures, perceived risk, and psychological distress in the general population in Wuhan. Exposure, no matter within a close physical distance or social network, was significantly linked to psychological distress. Perceived risk was not only directly associated with psychological distress but also mediated the effects of exposures. These findings highlight the necessity to include the psychosocial perspective in the emergency response and risk management system during the public health crisis, especially in the early stage when people are easy to panic. To cope with potential waves of the COVID-19 outbreak and similar pandemics in future, health education and crisis intervention focused on the perception among different social groups are expected to design and deliver based on valid empirical evidence.

## Data availability statement

The data analyzed in this study is subject to the following licenses/restrictions: data are available from the authors upon reasonable request and with permission of the project group of Community Life Survey among General Population in Wuhan during COVID-19 Outbreak. Requests to access these datasets should be directed to shizhilei2004@126.com.

## Author contributions

YL and ZS jointly framed the idea, developed the initial structure of the article, and finalized the manuscript. YL wrote the first draft. ZS revised the draft. LL took part in the revision of the introduction, methods, and discussion. All authors contributed to the article and approved the submitted version.

## Funding

YL was supported by the Youth Project of Social Sciences Foundation of Jiangsu Province (no. 19SHC001).

## Conflict of interest

The authors declare that the research was conducted in the absence of any commercial or financial relationships that could be construed as a potential conflict of interest.

## Publisher’s note

All claims expressed in this article are solely those of the authors and do not necessarily represent those of their affiliated organizations, or those of the publisher, the editors and the reviewers. Any product that may be evaluated in this article, or claim that may be made by its manufacturer, is not guaranteed or endorsed by the publisher.
